# First records of non-native species *Callitrichedeflexa* (Plantaginaceae), which was previously misidentified as *C.terrestris* in Japan

**DOI:** 10.3897/BDJ.12.e115142

**Published:** 2024-01-15

**Authors:** Hiroyuki Koga, Yuki Doll, Wataru Ohnishi, Hirokazu Tsukaya

**Affiliations:** 1 The University of Tokyo, Tokyo, Japan The University of Tokyo Tokyo Japan; 2 Kanagawa Prefectural Museum of Natural History, Odawara, Kanagawa, Japan Kanagawa Prefectural Museum of Natural History Odawara, Kanagawa Japan

**Keywords:** *Callitrichedeflexa*, *Callitricheterrestris*, non-native species, new record, Japan

## Abstract

**Background:**

The cosmopolitan genus *Callitriche* (Plantaginaceae) is a clade of small herbaceous plants that encompasses terrestrial and aquatic species. In Japan, six *Callitriche* species have been identified: four native and two naturalised species. *Callitricheterrestris*, a naturalised terrestrial species, was first reported in 1984 in Kanagawa Prefecture and it is thriving today.

**New information:**

We report the presence of a new naturalised terrestrial species, *Callitrichedeflexa*, which has been previously misidentified as *C.terrestris* because of its similar morphology. *Callitrichedeflexa* can be distinguished from *C.terrestris* through genetic differences and distinct morphological traits, such as longer pedicels. Re-examination of herbarium specimens in the Kanagawa Prefectural Museum of Natural History confirmed that most of the specimens labelled as *C.terrestris*, including voucher specimens from the original report, were indeed *C.terrestris*, but a few were *C.deflexa*. We also noted that the plants referred to as “*C.terrestris*” in our previous developmental studies should be corrected to *C.deflexa*.

## Introduction

The genus *Callitriche* L. belongs to the family Plantaginaceae and is composed of approximately 50–75 species (Lansdown 2008). The genus comprises primarily small, herbaceous species, many of which are aquatic in nature. *Callitriche* is often considered a challenging taxon to identify because of substantial variations in gross morphology in response to different environments, as well as an extremely simplified flower morphology (Lansdown 2008). Like many aquatic plants, some *Callitriche* species exhibit significant morphological variations in leaves and stems even within a single individual, as they are influenced by the aquatic habitat (Deschamp and Cooke 1985, Jones 1955, Koga et al. 2020). Therefore, flower and fruit characteristics become especially important for the identification of different *Callitriche* species. However, the *Callitriche* flower is achlamydeous and diclinous, consisting of only a carpel or stamen and lacking showy petals and sepals (Erbar and Leins 2004). These organs are often microscopic, making taxonomically relevant traits difficult to discern. Overall, these characteristics contribute to the difficulty in the accurate identification of *Callitriche* members.

In Japan, four native *Callitriche* species are currently recognised: *Callitrichehermaphroditica* L., *Callitrichejaponica* Engelm. ex Hegelmaier, *Callitrichepalustris* L. and *Callitrichefuscicarpa* Lansdown (Lansdown 2006b). In addition, *Callitricheterrestris* Muhl. ex Raf. (Katsuyama and Kawasumi 1999) and *Callitrichestagnalis* Scop. (Morita and Lee 1998) have been reported as naturalised species (Uemura et al. 2015). *Callitricheterrestris* is native to North and South America (Lansdown and Hassemer 2021) and it has been reported as a non-native species in Europe (Lansdown 2006a, Lansdown 2008, Lansdown et al. 2017b). *Callitrichestagnalis* is an aquatic species native to Europe and it is also a widespread alien species in various other regions worldwide, such as North and South America (Philbrick and Jansen 1991, Lansdown 2009, Hassemer and Lansdown 2018) and Australia (Bean 2007, Lansdown 2022).

A recent molecular phylogenetic study of *Callitriche* species, including Japanese specimens of some of the species mentioned above, revealed that *C.japonica* is a sister of the other major members of this genus, whereas *C.hermaphroditica*, *C.stagnalis* and *C.palustris* belong to distinct clades (Ito et al. 2017). The phylogenetic position of *C.terrestris* found in Japan has not yet been examined. On the basis of these phylogenetic relationships, several comparative developmental studies with the *Callitriche* species found in Japan have been conducted by us (Doll et al. 2021a, Doll et al. 2021b, Koga et al. 2021). To date, only *C.japonica* and *C.terrestris* have been recognised as terrestrial *Callitriche* species in Japan. Katsuyama and Kawasumi (1999) distinguished *C.terrestris* from *C.japonica* on the basis of its spatulate leaf shape, presence of pedicels and less pronounced fruit wings. According to these key traits, terrestrial *Callitriche* specimens from various regions of Japan, including those used in our studies, were identified as *C.terrestris*. However, laboratory cultivation and observations have shown us that at least the specimens used in our experiments had morphological traits that differed slightly from those originally described by Katsuyama and Kawasumi (1999). Therefore, we conducted a taxonomic re-examination of the plants regarded as *C.terrestris* by collecting them from several localities in Japan as well as observing herbarium specimens. Our investigations revealed *Callitrichedeflexa* A. Braun as a previously misidentified and non-native terrestrial species in Japan.

## Materials and methods

### Plant collection and morphological observations

Terrestrial *Callitriche* specimens were collected from various locations, such as Hyogo (HN2), Osaka (OH1), Fukuoka (FF2), Ibaraki (IbTkb1 and IbTcu1), Tokyo (TKoi1) and Kanagawa (KKmz1, KHd1, KHsm1) Prefectures (Table 1). The plant strain from Hyogo Prefecture (HN2) has been previously used in developmental biology studies (Doll et al. 2021a, Doll et al. 2021b, Koga et al. 2021). Some specimens co-occurred with *C.japonica*, but they could be easily distinguished by their shoot forms and fruit morphologies (Katsuyama 2018). The localities of KKmz1 and KHsm1 in Kanagawa Prefecture matched with the information of voucher specimens for *C.terrestris*, M. Matsumoto NA0112762 (KPM) and C. Hasekura NA0112764 (KPM), respectively (Katsuyama and Kawasumi 1999).

To observe the flower and fruit morphologies, the collected plants were cultured in soil (Mizukusa-ichiban sand, GEX, Japan) in a growth chamber with a long-day condition (16 h light and 8 h dark) at 22˚C. Some specimens were plants that were inbred for 1–5 generations after collection. The voucher specimens were deposited to Herbarium of the Department of Botany, University of Tokyo (TI; Table 1). Herbarium collections of Kanagawa Prefectural Museum of Natural History (KPM) were also examined for both morphological and genetic investigations (Suppl. material 1).

### DNA extraction and amplification of barcoding regions

To obtain genomic DNA for analysis, we extracted DNA from the shoot tips of herbarium specimens and living plants. For herbarium specimens, we first homogenised the dry samples by using TissueLyser II (Qiagen, Hilden, Germany) with zirconia beads and then performed DNA extraction by using the cetyl trimethyl ammonium bromide method, followed by purification with AMPure XP beads (Beckman Coulter, Brea, California, USA). For fresh samples, we used a DNeasy Plant Mini Kit (Qiagen). We amplified *matK* and *rbcL* regions of the extracted DNA by using PCR with specific primer sets: matK_390_F (5′-CGATCTATTCATTCAATATTTC-3′) and matK_1326R (5′-TCTAGCACACGAAAGTCGAAGT-3′) (Cuénoud et al. 2002); rbcL_5F (5′-ACCACAAACAGARACTAAAGC-3′) and rbcL_1210R (5′-AAGGRTGYCCTAAAGTTCCTCC-3′) (Les et al. 1993, Philbrick and Les 2000). In cases where PCR with the primer sets for fresh samples was unsuccessful for herbarium samples, we designed a new primer set that specifically targeted a short (199 bp) region of *matK* in the examined species: matK_497F (5′-ATTGGGTAAAAGATGCCTCTTCTTTGC-3′) and matK_695R (5′-TGAGAAGATTGGTTACGTAGAAAGACG-3′). We performed PCR with PrimeSTAR GXL Polymerase (Takara Bio, Shiga, Japan) or Ex Premier Polymerase (Takara Bio) and purified the PCR products with AMPure XP beads (Beckman Coulter). Sanger sequencing was used to sequence the purified products with the primers used for PCR. The resulting sequences were deposited in NCBI/DDBJ/EBI (accession numbers listed in Table 2 and Table 3).

### Phylogenetic analysis

To evaluate the phylogenetic positions of the samples, rbcL and matK sequences of *Callitriche* and some outgroup species were obtained from NCBI/DDBJ/EBI. The accession numbers are listed in Suppl. material 2. We used the data from Ito et al. (2017), which provided both *rbcL* and *matK* sequences from a broad range of *Callitriche* species. DNA alignment was conducted by MAFFT (Katoh and Standley 2013). The Maximum Likelihood tree was reconstructed using concatenated sequences of *matK* and *rbcL* by IQ-TREE 2 (Minh et al. 2020). The confidence values of each node were calculated using both ultrafast bootstrapping and Shimodaira–Hasegawa-like approximate likelihood ratio test (Hoang et al. 2017).

## Taxon treatments

### Callitriche deflexa

A. Braun, 1846

D7B8CE9C-F2DC-52CE-A403-7975D2715EB1

ナガエアワゴケ Nagae-awagoke (terrestrial water-starwort with long pedicels) (nov.); broad-fruited water-starwort (Lansdown 2008).

#### Materials

- **Type status:** Other material. **Occurrence:** occurrenceDetails: http://api.gbif.org/v1/occurrence/2244423416; catalogNumber: KPM-NA0208310; individualCount: 1; occurrenceStatus: present; occurrenceID: F529E51B-CE6E-52A8-8735-4A134E3B505C; **Taxon:** scientificName: Callitrichedeflexa A. Braun; kingdom: Plantae; phylum: Tracheophyta; class: Magnoliopsida; order: Lamiales; family: Plantaginaceae; genus: Callitriche; specificEpithet: deflexa; taxonRank: species; vernacularName: ナガエアワゴケ; taxonomicStatus: accepted; **Location:** higherGeography: Japan|Kanagawa Prefecture|Yamato City|Minamirinkan; continent: ASIA; country: Japan; countryCode: JP; stateProvince: Kanagawa Prefecture; county: Yamato City; locality: Minamirinkan; decimalLatitude: 35.490755; decimalLongitude: 139.440551; geodeticDatum: WGS84; **Event:** eventDate: 2016-06-08T00:00:00; year: 2016; month: 6; day: 8; **Record Level:** type: text; modified: 2019-02-28T00:00:00.000+00:00; language: en; institutionID: institutionID; collectionID: collectionID; institutionCode: KPM; collectionCode: NA; basisOfRecord: PreservedSpecimen
- **Type status:** Other material. **Occurrence:** occurrenceDetails: http://api.gbif.org/v1/occurrence/3324114555; catalogNumber: KPM-NA0203540; individualCount: 1; occurrenceStatus: present; occurrenceID: D34E21E0-9C75-5605-985C-19F62C0EDA26; **Taxon:** scientificName: Callitrichedeflexa A. Braun; kingdom: Plantae; phylum: Tracheophyta; class: Magnoliopsida; order: Lamiales; family: Plantaginaceae; genus: Callitriche; specificEpithet: deflexa; taxonRank: species; vernacularName: ナガエアワゴケ; taxonomicStatus: accepted; **Location:** higherGeography: Japan|Mie Prefecture|Yokkaichi City; continent: ASIA; country: Japan; countryCode: JP; stateProvince: Mie Prefecture; county: Yokkaichi City; decimalLatitude: 34.98294; decimalLongitude: 136.66162; geodeticDatum: WGS84; **Event:** eventDate: 2011-05-30T00:00:00; year: 2011; month: 5; day: 30; **Record Level:** type: text; modified: 2020-11-27T00:00:00.000+00:00; language: en; institutionID: institutionID; collectionID: collectionID; institutionCode: KPM; collectionCode: NA; basisOfRecord: PreservedSpecimen
- **Type status:** Other material. **Occurrence:** occurrenceDetails: http://api.gbif.org/v1/occurrence/3324109531; catalogNumber: KPM-NA0202813; individualCount: 1; occurrenceStatus: present; occurrenceID: D9FE4257-7A60-5D82-968A-2A6D22810830; **Taxon:** scientificName: Callitrichedeflexa A. Braun; kingdom: Plantae; phylum: Tracheophyta; class: Magnoliopsida; order: Lamiales; family: Plantaginaceae; genus: Callitriche; specificEpithet: deflexa; taxonRank: species; vernacularName: ナガエアワゴケ; taxonomicStatus: accepted; **Location:** higherGeography: Japan|Osaka Prefecture|Hannan City|Yamanakadani; continent: ASIA; country: Japan; countryCode: JP; stateProvince: Osaka Prefecture; county: Hannan City; locality: Yamanakadani; decimalLatitude: 34.32601; decimalLongitude: 135.27158; geodeticDatum: WGS84; **Event:** eventDate: 2013-04-09T00:00:00; year: 2013; month: 4; day: 9; **Record Level:** type: text; modified: 2020-11-27T00:00:00.000+00:00; language: en; institutionID: institutionID; collectionID: collectionID; institutionCode: KPM; collectionCode: NA; basisOfRecord: PreservedSpecimen
- **Type status:** Other material. **Occurrence:** occurrenceDetails: http://api.gbif.org/v1/occurrence/1829883645; catalogNumber: KPM-NA0116625; individualCount: 1; occurrenceStatus: present; occurrenceID: 9B6F284A-FE87-5EAC-8C63-3EACA2E427AC; **Taxon:** scientificName: Callitrichedeflexa A. Braun; kingdom: Plantae; phylum: Tracheophyta; class: Magnoliopsida; order: Lamiales; family: Plantaginaceae; genus: Callitriche; specificEpithet: deflexa; taxonRank: species; vernacularName: ナガエアワゴケ; taxonomicStatus: accepted; **Location:** higherGeography: Japan|Okinawa Prefecture|Nago-shi; continent: ASIA; country: Japan; countryCode: JP; stateProvince: Okinawa Prefecture; county: Nago-shi; decimalLatitude: 26.591944; decimalLongitude: 127.977222; geodeticDatum: WGS84; **Event:** eventDate: 1999-03-09T00:00:00; year: 1999; month: 3; day: 9; **Record Level:** type: text; modified: 2013-07-01T00:00:00.000+00:00; language: en; institutionID: institutionID; collectionID: collectionID; institutionCode: KPM; collectionCode: NA; basisOfRecord: PreservedSpecimen
- **Type status:** Other material. **Occurrence:** catalogNumber: TI00256641; occurrenceID: D2A453DF-5FC9-53BC-BB0B-8D95B7D2A34B; **Taxon:** scientificName: Callitrichedeflexa A. Braun; kingdom: Plantae; phylum: Tracheophyta; class: Magnoliopsida; order: Lamiales; family: Plantaginaceae; genus: Callitriche; specificEpithet: deflexa; taxonRank: species; vernacularName: ナガエアワゴケ; taxonomicStatus: accepted; **Location:** continent: Asia; country: Japan; countryCode: JP; stateProvince: Hyogo; county: Nishinomiya-shi; decimalLatitude: 34.7695; decimalLongitude: 135.3653; geodeticDatum: WGS84; **Event:** eventDate: 2015-09-27T00:00:00; year: 2015; month: 9; day: 27; **Record Level:** language: en; institutionCode: TI; basisOfRecord: PreservedSpecimen
- **Type status:** Other material. **Occurrence:** catalogNumber: TI00256643; recordedBy: Hiroyuki Koga; occurrenceID: 41DA7F8B-F3FC-5D4A-805F-91BA171BB6FE; **Taxon:** scientificName: Callitrichedeflexa A. Braun; kingdom: Plantae; phylum: Tracheophyta; class: Magnoliopsida; order: Lamiales; family: Plantaginaceae; genus: Callitriche; specificEpithet: deflexa; taxonRank: species; vernacularName: ナガエアワゴケ; taxonomicStatus: accepted; **Location:** continent: Asia; country: Japan; countryCode: JP; stateProvince: Osaka; county: Hirakata-shi; decimalLatitude: 34.8176; decimalLongitude: 135.6701; geodeticDatum: WGS84; **Event:** eventDate: 2015-09-20T00:00:00; year: 2015; month: 9; day: 20; **Record Level:** language: en; institutionCode: TI; basisOfRecord: PreservedSpecimen
- **Type status:** Other material. **Occurrence:** catalogNumber: TI00256647; recordedBy: Hiroyuki Koga; individualCount: 1; occurrenceID: 45915E75-EB4B-5FF1-9108-5C4EFB15EEB5; **Taxon:** scientificName: Callitrichedeflexa A. Braun; kingdom: Plantae; phylum: Tracheophyta; class: Magnoliopsida; order: Lamiales; family: Plantaginaceae; genus: Callitriche; specificEpithet: deflexa; taxonRank: species; vernacularName: ナガエアワゴケ; taxonomicStatus: accepted; **Location:** continent: Asia; country: Japan; countryCode: JP; stateProvince: Fukuoka; county: Fukuoka-shi; decimalLatitude: 33.5808; decimalLongitude: 130.3928; geodeticDatum: WGS84; **Event:** eventDate: 2020-02-02T00:00:00; year: 2020; month: 2; day: 2; **Record Level:** language: en; institutionCode: TI; basisOfRecord: PreservedSpecimen
- **Type status:** Other material. **Occurrence:** catalogNumber: TI00256639; recordedBy: Hiroyuki Koga; individualCount: 1; occurrenceID: A29E5A5F-72E2-5C91-B4E2-0D6D9769F623; **Taxon:** scientificName: Callitrichedeflexa A. Braun; kingdom: Plantae; phylum: Tracheophyta; class: Magnoliopsida; order: Lamiales; family: Plantaginaceae; genus: Callitriche; specificEpithet: deflexa; taxonRank: species; vernacularName: ナガエアワゴケ; taxonomicStatus: accepted; **Location:** continent: Asia; country: Japan; countryCode: JP; stateProvince: Ibaraki; county: Tsukuba-shi; decimalLatitude: 36.0625; decimalLongitude: 140.0799; geodeticDatum: WGS84; **Event:** eventDate: 2022-02-25T00:00:00; year: 2022; month: 2; day: 25; **Record Level:** language: en; institutionCode: TI; basisOfRecord: PreservedSpecimen
- **Type status:** Other material. **Occurrence:** catalogNumber: TI00256640; recordedBy: Hiroyuki Koga; individualCount: 1; occurrenceID: EBADE9B7-DA51-51A6-AE56-A6240EE2FA31; **Taxon:** scientificName: Callitrichedeflexa A. Braun; kingdom: Plantae; phylum: Tracheophyta; class: Magnoliopsida; order: Lamiales; family: Plantaginaceae; genus: Callitriche; specificEpithet: deflexa; taxonRank: species; vernacularName: ナガエアワゴケ; taxonomicStatus: accepted; **Location:** continent: Asia; country: Japan; countryCode: JP; stateProvince: Kanagawa; county: Yokohama-shi; municipality: Hodogaya-ku; decimalLatitude: 35.4440; decimalLongitude: 139.5969; geodeticDatum: WGS84; **Event:** eventDate: 2022-03-09T00:00:00; year: 2022; month: 3; day: 9; **Record Level:** language: en; institutionCode: TI; basisOfRecord: PreservedSpecimen
- **Type status:** Other material. **Occurrence:** catalogNumber: TI00256646; recordedBy: Yuki Doll; individualCount: 1; occurrenceID: F86F621B-9757-58B9-A678-CE63CF60553E; **Taxon:** scientificName: Callitrichedeflexa A. Braun; kingdom: Plantae; phylum: Tracheophyta; class: Magnoliopsida; order: Lamiales; family: Plantaginaceae; genus: Callitriche; specificEpithet: deflexa; taxonRank: species; vernacularName: ナガエアワゴケ; taxonomicStatus: accepted; **Location:** continent: Asia; country: Japan; countryCode: JP; stateProvince: Tokyo; county: Bunkyo-ku; municipality: Koishikawa; decimalLatitude: 35.7130; decimalLongitude: 139.7510; geodeticDatum: WGS84; **Event:** eventDate: 2022-03-06T00:00:00; year: 2022; month: 3; day: 6; **Record Level:** language: en; institutionCode: TI; basisOfRecord: PreservedSpecimen

#### Distribution

Native to Central South America: southern Brazil, Paraguay, Uruguay and northern Argentina (Fassett 1951, Lansdown and Hassemer 2021). Naturalised in Australia (Lansdown and Hassemer 2021, Lansdown 2022), Europe (Lansdown 2006a, Lansdown 2008, Raab-Straube 2014), Africa (Lansdown et al. 2017a, Lansdown et al. 2022), Taiwan (Chen et al. 2013) and Japan (newly reported here).

### Callitriche terrestris

Muhl. ex Raf., 1808

21103395-D5AC-5E00-9074-FE2C85643ADB

アメリカアワゴケ Amerika-awagoke (American terrestrial water-starwort) (Katsuyama and Kawasumi 1999); terrestrial water-starwort (Lansdown 2008).

#### Materials

- **Type status:** Other material. **Occurrence:** occurrenceDetails: http://api.gbif.org/v1/occurrence/187978085; catalogNumber: KPM-NA1004382; individualCount: 1; occurrenceStatus: PRESENT; occurrenceID: 9FE12147-770D-5906-9AD9-DBDBBA5C6169; **Taxon:** scientificName: Callitricheterrestris Muhl. ex Raf.; kingdom: Plantae; phylum: Tracheophyta; class: Magnoliopsida; order: Lamiales; family: Plantaginaceae; genus: Callitriche; specificEpithet: terrestris; taxonRank: species; vernacularName: アメリカアワゴケ; taxonomicStatus: accepted; **Location:** higherGeography: Japan|Kanagawa|Ebina-shi|Kawaraguchi,Ebina City, Kawara-guti; continent: ASIA; country: Japan; countryCode: JP; stateProvince: Kanagawa; county: Ebina-shi; locality: Kawaraguchi,Ebina City, Kawara-guti; decimalLatitude: 35.449095; decimalLongitude: 139.378057; geodeticDatum: WGS84; **Event:** eventDate: 1984-06-11T00:00:00; year: 1984; month: 6; day: 11; **Record Level:** type: text; modified: 2007-05-09T00:00:00.000+00:00; language: en; institutionID: institutionID; collectionID: collectionID; institutionCode: KPM; collectionCode: NA; basisOfRecord: PreservedSpecimen
- **Type status:** Other material. **Occurrence:** occurrenceDetails: http://api.gbif.org/v1/occurrence/187978079; catalogNumber: KPM-NA1004375; individualCount: 1; occurrenceStatus: PRESENT; occurrenceID: CB3D07CB-089A-51F4-AE93-A81FA3DB55E0; **Taxon:** scientificName: Callitricheterrestris Muhl. ex Raf.; kingdom: Plantae; phylum: Tracheophyta; class: Magnoliopsida; order: Lamiales; family: Plantaginaceae; genus: Callitriche; specificEpithet: terrestris; taxonRank: species; vernacularName: アメリカアワゴケ; taxonomicStatus: accepted; **Location:** higherGeography: Japan|Kanagawa|Yokohama-shi Nishi-ku|Motokubo-cho,Yokohama City, Nishi-ku, Motokubo-cho; continent: ASIA; country: Japan; countryCode: JP; stateProvince: Kanagawa; county: Yokohama-shi Nishi-ku; locality: Motokubo-cho,Yokohama City, Nishi-ku, Motokubo-cho; decimalLatitude: 35.440766; decimalLongitude: 139.60304; geodeticDatum: WGS84; **Event:** eventDate: 1985-05-22T00:00:00; year: 1985; month: 5; day: 22; **Record Level:** type: text; modified: 2007-05-09T00:00:00.000+00:00; language: en; institutionID: institutionID; collectionID: collectionID; institutionCode: KPM; collectionCode: NA; basisOfRecord: PreservedSpecimen
- **Type status:** Other material. **Occurrence:** occurrenceDetails: http://api.gbif.org/v1/occurrence/187978076; catalogNumber: KPM-NA1004372; individualCount: 1; occurrenceStatus: PRESENT; occurrenceID: D5778624-3727-5AC0-B429-9B19F1F920EB; **Taxon:** scientificName: Callitricheterrestris Muhl. ex Raf.; kingdom: Plantae; phylum: Tracheophyta; class: Magnoliopsida; order: Lamiales; family: Plantaginaceae; genus: Callitriche; specificEpithet: terrestris; taxonRank: species; vernacularName: アメリカアワゴケ; taxonomicStatus: accepted; **Location:** higherGeography: Japan|Kanagawa|Yokohama-shi Kanagawa-ku|Yokohama City, Kanagawa-ku, Mitsuzawa Park; continent: ASIA; country: Japan; countryCode: JP; stateProvince: Kanagawa; county: Yokohama-shi Kanagawa-ku; locality: Yokohama City, Kanagawa-ku, Mitsuzawa Park; decimalLatitude: 35.474095; decimalLongitude: 139.603039; geodeticDatum: WGS84; **Event:** eventDate: 1985-07-04T00:00:00; year: 1985; month: 7; day: 4; **Record Level:** type: text; modified: 2007-05-09T00:00:00.000+00:00; language: en; institutionID: institutionID; collectionID: collectionID; institutionCode: KPM; collectionCode: NA; basisOfRecord: PreservedSpecimen
- **Type status:** Other material. **Occurrence:** occurrenceDetails: http://api.gbif.org/v1/occurrence/388800331; catalogNumber: KPM-NA0112761; individualCount: 1; occurrenceStatus: PRESENT; occurrenceID: 3832C2BA-8F0D-5052-AC76-C778571D0A67; **Taxon:** scientificName: Callitricheterrestris Muhl. ex Raf.; kingdom: Plantae; phylum: Tracheophyta; class: Magnoliopsida; order: Lamiales; family: Plantaginaceae; genus: Callitriche; specificEpithet: terrestris; taxonRank: species; vernacularName: アメリカアワゴケ; taxonomicStatus: accepted; **Location:** higherGeography: Japan|Kanagawa Prefecture|Yokohama-shi, Minami-ku; continent: ASIA; country: Japan; countryCode: JP; stateProvince: Kanagawa Prefecture; county: Yokohama-shi, Minami-ku; decimalLatitude: 35.432434; decimalLongitude: 139.628038; geodeticDatum: WGS84; **Event:** eventDate: 1997-06-13T00:00:00; year: 1997; month: 6; day: 13; **Record Level:** type: text; modified: 2013-07-05T00:00:00.000+00:00; language: en; institutionID: institutionID; collectionID: collectionID; institutionCode: KPM; collectionCode: NA; basisOfRecord: PreservedSpecimen
- **Type status:** Other material. **Occurrence:** occurrenceDetails: http://api.gbif.org/v1/occurrence/388800334; catalogNumber: KPM-NA0112764; individualCount: 1; occurrenceStatus: PRESENT; occurrenceID: 3E59BC2D-4F3A-5AD8-B143-B2CA60D17C36; **Taxon:** scientificName: Callitricheterrestris Muhl. ex Raf.; kingdom: Plantae; phylum: Tracheophyta; class: Magnoliopsida; order: Lamiales; family: Plantaginaceae; genus: Callitriche; specificEpithet: terrestris; taxonRank: species; vernacularName: アメリカアワゴケ; taxonomicStatus: accepted; **Location:** higherGeography: Japan|Kanagawa Prefecture|Sagamihara-shi; continent: ASIA; country: Japan; countryCode: JP; stateProvince: Kanagawa Prefecture; county: Sagamihara-shi; decimalLatitude: 35.607403; decimalLongitude: 139.340555; geodeticDatum: WGS84; **Event:** eventDate: 1998-06-18T00:00:00; year: 1998; month: 6; day: 18; **Record Level:** type: text; modified: 2013-07-05T00:00:00.000+00:00; language: en; institutionID: institutionID; collectionID: collectionID; institutionCode: KPM; collectionCode: NA; basisOfRecord: PreservedSpecimen
- **Type status:** Other material. **Occurrence:** occurrenceDetails: http://api.gbif.org/v1/occurrence/388800332; catalogNumber: KPM-NA0112762; individualCount: 1; occurrenceStatus: PRESENT; occurrenceID: FA544454-3E6D-5B78-B8BF-6917C4F45006; **Taxon:** scientificName: Callitricheterrestris Muhl. ex Raf.; kingdom: Plantae; phylum: Tracheophyta; class: Magnoliopsida; order: Lamiales; family: Plantaginaceae; genus: Callitriche; specificEpithet: terrestris; taxonRank: species; vernacularName: アメリカアワゴケ; taxonomicStatus: accepted; **Location:** higherGeography: Japan|Kanagawa Prefecture|Sagamihara-shi; continent: ASIA; country: Japan; countryCode: JP; stateProvince: Kanagawa Prefecture; county: Sagamihara-shi; decimalLatitude: 35.549079; decimalLongitude: 139.365555; geodeticDatum: WGS84; **Event:** eventDate: 1998-06-01T00:00:00; year: 1998; month: 6; day: 1; **Record Level:** type: text; modified: 2013-07-05T00:00:00.000+00:00; language: en; institutionID: institutionID; collectionID: collectionID; institutionCode: KPM; collectionCode: NA; basisOfRecord: PreservedSpecimen
- **Type status:** Other material. **Occurrence:** occurrenceDetails: http://api.gbif.org/v1/occurrence/245530607; catalogNumber: KPM-NA1104918; individualCount: 1; occurrenceStatus: PRESENT; occurrenceID: 24E5E4FE-575E-557B-A18E-D84585642D20; **Taxon:** scientificName: Callitricheterrestris Muhl. ex Raf.; kingdom: Plantae; phylum: Tracheophyta; class: Magnoliopsida; order: Lamiales; family: Plantaginaceae; genus: Callitriche; specificEpithet: terrestris; taxonRank: species; vernacularName: アメリカアワゴケ; taxonomicStatus: accepted; **Location:** higherGeography: Japan|Kanagawa Prefecture|Yokohama-shi, Hodogaya-ku; continent: ASIA; country: Japan; countryCode: JP; stateProvince: Kanagawa Prefecture; county: Yokohama-shi, Hodogaya-ku; decimalLatitude: 35.449098; decimalLongitude: 139.590541; geodeticDatum: WGS84; **Event:** eventDate: 1992-06-27T00:00:00; year: 1992; month: 6; day: 27; **Record Level:** type: text; modified: 2010-06-09T00:00:00.000+00:00; language: en; institutionID: institutionID; collectionID: collectionID; institutionCode: KPM; collectionCode: NA; basisOfRecord: PreservedSpecimen
- **Type status:** Other material. **Occurrence:** occurrenceDetails: http://api.gbif.org/v1/occurrence/2244423413; catalogNumber: KPM-NA0208327; individualCount: 1; occurrenceStatus: PRESENT; occurrenceID: CD68C85C-CE86-5966-AC57-5648D008283F; **Taxon:** scientificName: Callitricheterrestris Muhl. ex Raf.; kingdom: Plantae; phylum: Tracheophyta; class: Magnoliopsida; order: Lamiales; family: Plantaginaceae; genus: Callitriche; specificEpithet: terrestris; taxonRank: species; vernacularName: アメリカアワゴケ; taxonomicStatus: accepted; **Location:** higherGeography: Japan|Kanagawa-Ken|Sagamihara-shi, Minami-ku|Shimomizo; continent: ASIA; country: Japan; countryCode: JP; stateProvince: Kanagawa-Ken; county: Sagamihara-shi, Minami-ku; locality: Shimomizo; decimalLatitude: 35.524084; decimalLongitude: 139.378055; geodeticDatum: WGS84; **Event:** eventDate: 2004-04-20T00:00:00; year: 2004; month: 4; day: 20; **Record Level:** type: text; modified: 2019-02-28T00:00:00.000+00:00; language: en; institutionID: institutionID; collectionID: collectionID; institutionCode: KPM; collectionCode: NA; basisOfRecord: PreservedSpecimen
- **Type status:** Other material. **Occurrence:** occurrenceDetails: http://api.gbif.org/v1/occurrence/388800487; catalogNumber: KPM-NA0162807; individualCount: 1; occurrenceStatus: PRESENT; occurrenceID: 84880289-CB0F-5ED8-AA84-0523BF49C19E; **Taxon:** scientificName: Callitricheterrestris Muhl. ex Raf.; kingdom: Plantae; phylum: Tracheophyta; class: Magnoliopsida; order: Lamiales; family: Plantaginaceae; genus: Callitriche; specificEpithet: terrestris; taxonRank: species; vernacularName: アメリカアワゴケ; taxonomicStatus: accepted; **Location:** higherGeography: Japan|Kanagawa Prefecture|Yokohama-shi, Izumi-ku; continent: ASIA; country: Japan; countryCode: JP; stateProvince: Kanagawa Prefecture; county: Yokohama-shi, Izumi-ku; decimalLatitude: 35.390771; decimalLongitude: 139.49055; geodeticDatum: WGS84; **Event:** eventDate: 2009-07-07T00:00:00; year: 2009; month: 7; day: 7; **Record Level:** type: text; modified: 2013-07-06T00:00:00.000+00:00; language: en; institutionID: institutionID; collectionID: collectionID; institutionCode: KPM; collectionCode: NA; basisOfRecord: PreservedSpecimen
- **Type status:** Other material. **Occurrence:** occurrenceDetails: http://api.gbif.org/v1/occurrence/388800461; catalogNumber: KPM-NA0136933; individualCount: 1; occurrenceStatus: PRESENT; occurrenceID: 015992B6-44B1-591F-8044-5AD8086A4196; **Taxon:** scientificName: Callitricheterrestris Muhl. ex Raf.; kingdom: Plantae; phylum: Tracheophyta; class: Magnoliopsida; order: Lamiales; family: Plantaginaceae; genus: Callitriche; specificEpithet: terrestris; taxonRank: species; vernacularName: アメリカアワゴケ; taxonomicStatus: accepted; **Location:** higherGeography: Japan|Kanagawa Prefecture|Yokohama-shi, Minami-ku; continent: ASIA; country: Japan; countryCode: JP; stateProvince: Kanagawa Prefecture; county: Yokohama-shi, Minami-ku; decimalLatitude: 35.432083; decimalLongitude: 139.576889; geodeticDatum: WGS84; **Event:** eventDate: 2009-07-07T00:00:00; year: 2009; month: 7; day: 7; **Record Level:** type: text; modified: 2013-07-05T00:00:00.000+00:00; language: en; institutionID: institutionID; collectionID: collectionID; institutionCode: KPM; collectionCode: NA; basisOfRecord: PRESERVED_SPECIMEN
- **Type status:** Other material. **Occurrence:** occurrenceDetails: http://api.gbif.org/v1/occurrence/1829916404; catalogNumber: KPM-NA0176499; individualCount: 1; occurrenceStatus: PRESENT; occurrenceID: 036F3363-A5A9-570E-993A-78A695F9BF97; **Taxon:** scientificName: Callitricheterrestris Muhl. ex Raf.; kingdom: Plantae; phylum: Tracheophyta; class: Magnoliopsida; order: Lamiales; family: Plantaginaceae; genus: Callitriche; specificEpithet: terrestris; taxonRank: species; vernacularName: アメリカアワゴケ; taxonomicStatus: accepted; **Location:** higherGeography: Japan|Kanagawa Prefecture|Hadano-shi|Minamiyana; continent: ASIA; country: Japan; countryCode: JP; stateProvince: Kanagawa Prefecture; county: Hadano-shi; locality: Minamiyana; decimalLatitude: 35.365769; decimalLongitude: 139.265567; geodeticDatum: WGS84; **Event:** eventDate: 2014-06-08T00:00:00; year: 2014; month: 6; day: 8; **Record Level:** type: text; modified: 2015-12-19T00:00:00.000+00:00; language: en; institutionID: institutionID; collectionID: collectionID; institutionCode: KPM; collectionCode: NA; basisOfRecord: PreservedSpecimen
- **Type status:** Other material. **Occurrence:** occurrenceDetails: http://api.gbif.org/v1/occurrence/2244425949; catalogNumber: KPM-NA0216556; individualCount: 1; occurrenceStatus: PRESENT; occurrenceID: 52C34102-9BAC-54BE-BCEA-A32688FA0A4A; **Taxon:** scientificName: Callitricheterrestris Muhl. ex Raf.; kingdom: Plantae; phylum: Tracheophyta; class: Magnoliopsida; order: Lamiales; family: Plantaginaceae; genus: Callitriche; specificEpithet: terrestris; taxonRank: species; vernacularName: アメリカアワゴケ; taxonomicStatus: accepted; **Location:** higherGeography: Japan|Kanagawa Prefecture|Yokohama City, Hodogaya-ku|Hoshikawa; continent: ASIA; country: Japan; countryCode: JP; stateProvince: Kanagawa Prefecture; county: Yokohama City, Hodogaya-ku; locality: Hoshikawa; decimalLatitude: 35.457431; decimalLongitude: 139.590541; geodeticDatum: WGS84; **Event:** eventDate: 2015-06-02T00:00:00; year: 2015; month: 6; day: 2; **Record Level:** type: text; modified: 2019-02-28T00:00:00.000+00:00; language: en; institutionID: institutionID; collectionID: collectionID; institutionCode: KPM; collectionCode: NA; basisOfRecord: PreservedSpecimen
- **Type status:** Other material. **Occurrence:** catalogNumber: TI00256644; recordedBy: Hiroyuki Koga; occurrenceID: C1754382-903E-52EE-90E8-46C7E3A26881; **Taxon:** scientificName: Callitricheterrestris Muhl. ex Raf.; kingdom: Plantae; phylum: Tracheophyta; class: Magnoliopsida; order: Lamiales; family: Plantaginaceae; genus: Callitriche; specificEpithet: terrestris; taxonRank: species; vernacularName: アメリカアワゴケ; taxonomicStatus: accepted; **Location:** continent: Asia; country: Japan; countryCode: JP; stateProvince: Kanagawa; county: Sagamihara-shi; municipality: Kamimizo; decimalLatitude: 35.548853; decimalLongitude: 139.370462; geodeticDatum: WGS84; **Event:** eventDate: 2022-05-02T00:00:00; year: 2022; month: 5; day: 2; **Record Level:** language: en; institutionCode: TI; basisOfRecord: PreservedSpecimen
- **Type status:** Other material. **Occurrence:** catalogNumber: TI00256645; recordedBy: Hiroyuki Koga; occurrenceID: D6172080-FA45-59E8-949D-DD1AB9A222EB; **Taxon:** scientificName: Callitricheterrestris Muhl. ex Raf.; kingdom: Plantae; phylum: Tracheophyta; class: Magnoliopsida; order: Lamiales; family: Plantaginaceae; genus: Callitriche; specificEpithet: terrestris; taxonRank: species; vernacularName: アメリカアワゴケ; taxonomicStatus: accepted; **Location:** continent: Asia; country: Japan; countryCode: JP; stateProvince: Kanagawa; county: Sagamihara-shi; municipality: Hoshimoto; decimalLatitude: 35.601606; decimalLongitude: 139.347401; geodeticDatum: WGS84; **Event:** eventDate: 2022-05-02T00:00:00; year: 2022; month: 5; day: 2; **Record Level:** language: en; institutionCode: TI; basisOfRecord: PreservedSpecimen
- **Type status:** Other material. **Occurrence:** catalogNumber: TI00256642; recordedBy: Hiroyuki Koga; occurrenceID: 4E8227EB-4953-538E-8541-0194F6E6CC47; **Taxon:** scientificName: Callitricheterrestris Muhl. ex Raf.; kingdom: Plantae; phylum: Tracheophyta; class: Magnoliopsida; order: Lamiales; family: Plantaginaceae; genus: Callitriche; specificEpithet: terrestris; taxonRank: species; vernacularName: アメリカアワゴケ; taxonomicStatus: accepted; **Location:** continent: Asia; country: Japan; countryCode: JP; stateProvince: Ibaraki; county: Tsuchiura-shi; locality: Ottonuma Park; decimalLatitude: 36.04474; decimalLongitude: 140.15070; geodeticDatum: WGS84; **Event:** eventDate: 2022-05-20T00:00:00; year: 2022; month: 5; day: 20; **Record Level:** language: en; institutionCode: TI; basisOfRecord: PreservedSpecimen

#### Distribution

Native to North and South America: United States, Mexico and South American countries (Fassett 1951, Lansdown 2009, Hassemer and Lansdown 2018, Lansdown and Hassemer 2021). Naturalised in Europe (Lansdown 2006a, Lansdown 2008, Lansdown et al. 2017b), Taiwan and Japan (Katsuyama and Kawasumi 1999).

## Identification Keys

### Keys to identification of *Callitriche* species recorded from Japan

**Table:** 

1	Bracts absent	2
–	Bract present	5
2	Ovate or spatulated leaves absent. Linear submerged leaves present	*C.hermaphroditica*
–	Ovate or spatulated leaves present. Linear submerged leaves absent	3
3	Fruits narrower at base than at apex; fruits subsessile	*C.japonica*
–	Base of fruit not narrowed; ripe fruits clearly pedicellate > 0.5 mm	4
4	Some pedicels > 1 mm; some nodes in which female and male flowers in one axil opposed by a non-flowering axil; ripe fruits clearly winged; some styles > 1 mm in length	*C.deflexa*
–	All pedicels ≤ 1 mm; when female and male flowers in a leaf axil, always more than one female flower and/or one male flower in the opposite axil; fruit wing unclear; style always ≤ 1 mm long, shorter than ripe fruit	*C.terrestris*
5	Fruits almost the same length and width, with wings on all edges	*C.stagnalis*
–	Fruits longer than wide	6
6	Ovate leaf venation simple; fruits clearly winged at the apex only or the wing at the apex distinctly wider than below or fruits unwinged; when fruit unwinged, bracts as long and wide as ripe fruits	*C.palustris*
–	Ovate leaf venation complex; fruits unwinged or only narrowly winged all around; bracts as long as, but narrower than ripe fruitsOvate leaf venation complex; fruits unwinged or only narrowly winged all around; bracts as long as, but narrower than ripe fruits	*C.fuscicarpa*

We provide identification keys for the species level. See Lansdown (2006b) for varieties of *C.palustris*.

## Analysis

### Morphological traits

**Figure 1.**
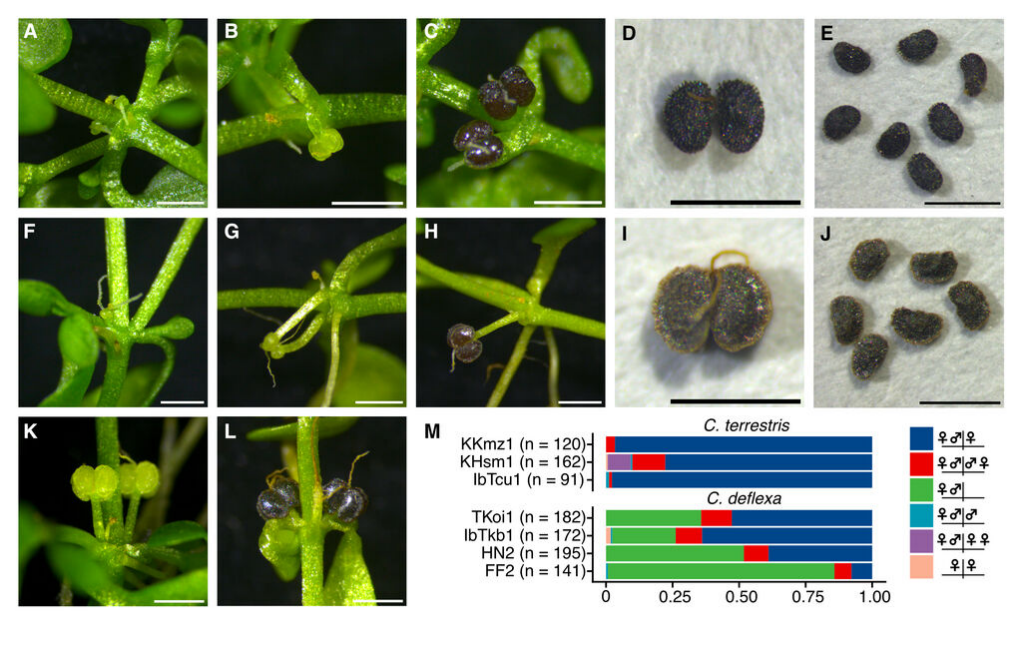
Flower and fruit morphologies of *C.terrestris* and *C.deflexa*. (A–E) *Callitricheterrestris* (KKmz1 and IbTcu1) and (F–L) *C.deflexa* (HN2 and FF2). **A** A node with two young female flowers and one male flower; **B** An immature fruit with a short deflexed pedicel; **C** A node with mature fruits; **D, I** Fully matured schizocarp abscised from the pedicel; **E, J** Mericarps; **F** A node with one young female flower and one male flower in an axil; **G** Young female flowers with elongated pedicels; **H** Mature fruit with an elongated pedicel; **K** A node with two female flowers and one male flower; **L** Mature fruits with short pedicels; **M** The proportion of flowering patterns of nodes. Scale bars: 1 mm.

On the basis of recent descriptions (Lansdown et al. 2017a, Lansdown et al. 2017b, Hassemer and Lansdown 2018, Lansdown and Hassemer 2021), morphological traits of the collected plants, particularly flowers and fruits, were examined. The plants were raised in a growth chamber and harvested before observation. The specimens were divided into two types. One type, represented by specimens KKmz1, KHsm1 and IbTcu1, displayed traits consistent with those of *C.terrestris*, as described in an original report from Japan (Katsuyama and Kawasumi 1999), including short pedicels (≤ 1 mm) that point downwards when the fruits mature (Fig. 1B, C). It is common to find one female and one male flower in an axil, with a solitary female flower in the opposite axil (Fig. 1A, M). The fruit wings are narrow and not easily noticeable until the fruit is fully mature and dry (Fig. 1C–E). The styles, which persist when the fruit is fully mature, are typically shorter than the fruit height (Fig. 1A–C). Mature fruits are blackish, 0.5–0.7 mm in length and 0.6–0.8 mm in width (Fig. 1D, E). These traits also match the description of *C.terrestris* in recent taxonomic papers from Europe and South America (Hassemer and Lansdown 2018, Lansdown and Hassemer 2021). *Callitricheturfosa* Bertero ex Hegelmaier, which was previously referred to as subspecies Callitricheterrestrissubsp.turfosa (Bertero ex Hegelm.) Bacigalupo and is reportedly very similar in morphology to *C.terrestris*, has a convex fruit surface (Lansdown and Hassemer 2021), whereas specimens KKmz1, KHsm1 and IbTcu1 have a relatively flat surface. Therefore, they were identified as *C.terrestris* rather than *C.turfosa*.

The other type of specimens showed a similar shoot appearance, but had different flower and fruit morphologies. These plants often produced fruits with elongated pedicels (>1 mm) usually directed downwards (Fig. 1G, H) and short pedicels were sometimes observed (Fig. 1K, L). Male and female flowers were often found on the same axil, whereas the opposite axil often lacked a flower (Fig. 1F, H, M). Mature fruits had clear wings on all sides (Fig. 1I, J). Styles were either erect or weakly recurved and persisted on fully mature fruits and were often longer than the fruit height (Fig. 1G, L). Mature fruits were brown, measuring 0.5–0.7 mm in length and 0.7–0.9 mm in width (Fig. 1I). These characteristics were consistent with the keys for *C.deflexa* in recent literature (Lansdown et al. 2017a, Hassemer and Lansdown 2018, Lansdown and Hassemer 2021), leading us to conclude that these specimens were *C.deflexa*.

**Figure 2.**
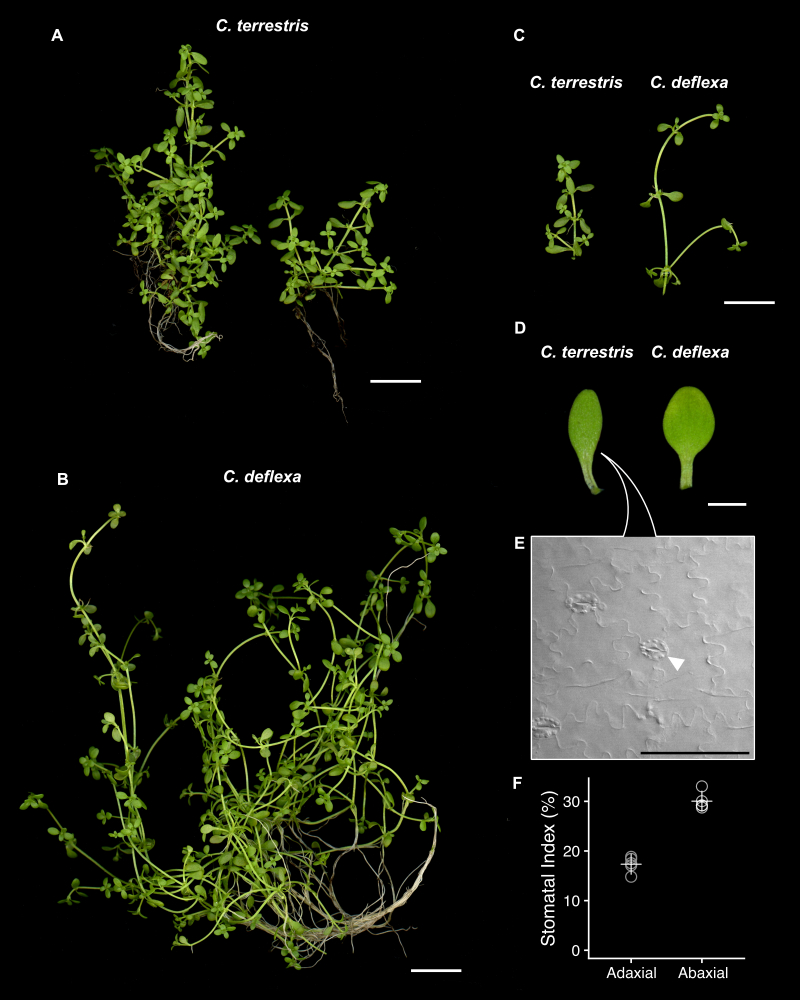
Shoot morphologies of *C.terrestris* and *C.deflexa*. **A** A whole-plant image of *C.terrestris*; **B** A whole plant image of *C.deflexa*; **C** Images of shoots; **D** Leaf shapes; **E** Abaxial epidermis of *C.terrestris* leaf. Guard cells indicated by an arrowhead are surrounded by three cells of heterogeneous size, suggesting that this lineage has undergone amplifying division; **F** Stomatal index (stomatal number per epidermal cell) on the adaxial and abaxial sides of *C.terrestris* leaves (n = 5 each). Circles indicate the values of each leaf and crosses indicate mean values. Scale bars: **A**–**C** 1 cm, **D** 1 mm, **E** 100 µm.

The shoots of both *C.terrestris* and *C.deflexa* are similar in appearance, but some differences become apparent when they are grown under the same conditions (Fig. 2). For example, the stem of *C.deflexa* tends to be longer and the mature leaves are rounder in shape when compared with *C.terrestris* (Fig. 2). These differences in shoot traits are stable when the plants are grown in a controlled environment, such as a growth chamber, but they may vary in different natural environments and are, thus, not suitable for rigid identification in the field. Previous studies have shown that the stomatal development of *C.deflexa* (denoted as *C.terrestris* in these papers) has some specific characteristics, such as amplifying division of stomatal lineage (Doll et al. 2021b) and stomata that differentiate on both sides of the leaf (amphistomy) (Doll et al. 2021a). Here, we confirmed that these characteristics were also shared by genuine *C.terrestris* (Fig. 2E, F).

Although the collected specimens showed differences in the traits described above, these traits were the keys to identifying *C.deflexa* and no traits could be used to identify *C.terrestris*. For example, flowering nodes in which a female flower is opposed by another female flower are also often observed in *C.deflexa* (Fig. 1G, K, M). Furthermore, the pedicel length of *C.deflexa* is variable and sometimes as short as that of *C.terrestris* (Fig. 1L). Thus, multiple traits of many flowers and fruits need to be comprehensively analysed for morphological identification.

### DNA sequence analysis

Next, we analysed DNA sequences to evaluate the genetic divergence between the two specimen types. According to previous studies that extensively examined the phylogenetic relationships of the genus *Callitriche* (Ito et al. 2017), *matK* and *rbcL* sequences were sequenced from the specimens. All sequences from specimens identified as *C.deflexa* on the basis of morphology were identical to *C.deflexa* analysed by Ito et al. (2017) (in the paper, this specimen was denoted as *Callitrichecompressa* N.E. Br. on the basis of the original identification, but the identification of the specimen was corrected to *C.deflexa* by Lansdown et al. (2017a)) (Tables 2, 3). In contrast, the rbcL sequence of a specimen assigned to *C.deflexa* by Philbrick and Les (2000) (Table 3) did not match the rbcL sequence of *C.deflexa*. As some traits of the specimen used by Philbrick and Les (2000) are inconsistent with the above-mentioned description, it is highly likely that the specimen is not *C.deflexa*, but a different species from the current taxonomic point of view. All three specimens morphologically identified as *C.terrestris* shared identical sequences, with 16 variant sites in *matK* and four variant sites in *rbcL* when compared with *C.deflexa* (Table 2 and Table 3). The *rbcL* sequence was identical to that of *C.terrestris* analysed by Philbrick and Les (2000).

**Figure 3.**
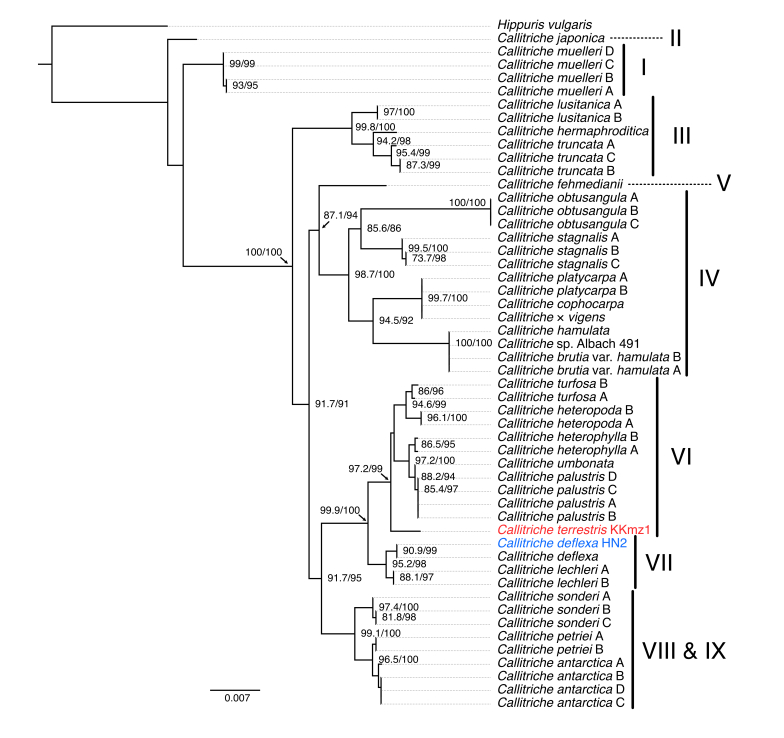
Phylogenetic tree of a plastid gene dataset (*matK* and *rbcL*). A Maximum Likelihood tree reconstructed by concatenated alignment of *matK* and *rbcL* DNA sequences. Node values represent Shimodaira–Hasegawa-like approximate likelihood ratio test (SH-aLRT) support (%)/ultrafast bootstrap (UFboot) support (%). Node values with either SH-aLRT support < 80% or UFboot support < 95% were omitted as they were not reasonably supported. Clade names (I to IX) were assigned according to Ito et al. (2017). Due to there being no sequence differences amongst the specimens obtained for this study, we used the sequence of only one specimen for each species.

As the phylogenetic position of *C.terrestris* was not examined even in previous broad phylogenetic analyses (Ito et al. 2017, Prančl et al. 2020), we performed a phylogenetic tree reconstruction by combining *matK* and *rbcL* sequences (Fig. 3). The result placed *C.terrestris* in clade VI, according to Ito et al. (2017). It is not a sister group to the morphologically similar *C.turfosa* and the results do not support the systematics that placed the two taxa as subspecies. However, the validity of the identification of specimens whose sequences were analysed as *C.turfosa* must be reviewed using the current taxonomic view. *Callitrichedeflexa* belonged to clade VII, which is consistent with the results of previous studies (Ito et al. 2017, Lansdown et al. 2017a).

### Re-examination of the herbarium specimens

**Figure 4.**
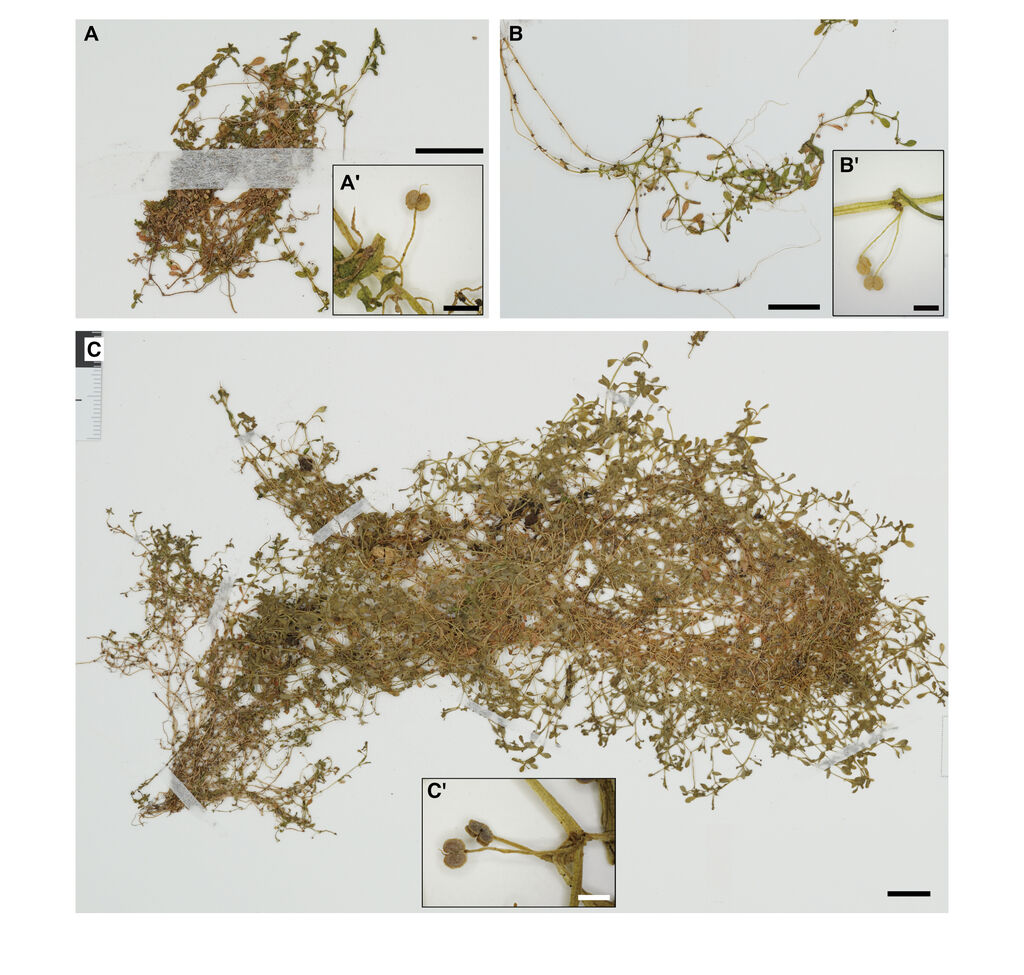
Herbarium specimens of *C.deflexa*. A representative specimen with a magnified image of the female flowers with elongated pedicels. **A** M. Matsumoto NA0116625 (KPM) from Okinawa Pref.; **B** M. Matsumoto NA0203540 (KPM) from Mie Pref.; **C** M. Matsumoto NA0208310 (KPM) from Kanagawa Pref. Scale bars: 1 cm for the whole plant panels and 1 mm for the magnified panels.

We re-examined herbarium specimens from KPM, which has the largest collection of specimens previously identified as *C.terrestris* in Japan. We confirmed that most of the herbarium specimens were *C.terrestris*, but some specimens showed the characteristics of *C.deflexa*: extensive variation in pedicel length, sometimes reaching more than 3 mm; clear wings on fruit edges (Fig. 4). This type of plant has been recorded in Kanagawa, Mie, Osaka and Okinawa Prefectures. The oldest specimen was collected in 1999 in Okinawa Prefecture, indicating that *C.deflexa* has been misidentified as *C.terrestris* in Japan for at least 24 years.

We also tried to amplify and sequence plastid DNA from some herbarium specimens deposited in KPM (Suppl. material 1). Due to the difficulties in DNA extraction from herbarium specimens, we could obtain only short fragments of *matK* sequences from limited specimens (Suppl. material 1). Nevertheless, we confirmed clear genotypic differences that were consistent with the morphological differences. All examined voucher specimens of NA1004372, NA1104918 and NA0112762; KPM and additional recently-collected specimens (NA0162807 and NA0216556; KPM) had the same sequences as *C.terrestris*, which we confirmed using the freshly-collected specimens. We also succeeded in obtaining amplicons from three out of four specimens that are probably *C.deflexa* on the basis of the morphological traits and found that they have sequences distinct from those of *C.terrestris*. Except for one single nucleotide variant (SNV) site (position 562) found in the specimens from Mie and Kanagawa, the sequences were identical to that of *C.deflexa* collected in this study (Table 2). In conclusion, on the basis of both morphology and genetics, two clearly different types of specimens were found in this study: *C.terrestris*, as identified previously and *C.deflexa*.

## Discussion

In this study, we found that two terrestrial *Callitriche* species have become naturalised in Japan: *C.terrestris* and *C.deflexa*. We propose the Japanese name “Nagae-awagoke” for *C.deflexa*, which means “terrestrial water-starwort with long pedicels,” on the basis of its morphological features. This is the first report of *C.deflexa* in Japan because it has often been misidentified as *C.terrestris* in the past. In fact, a book on naturalised plants in Japan has a page for *C.terrestris* with photos of plants that resemble *C.deflexa*, as well as true *C.terrestris* (Uemura et al. 2015). Additionally, plants that have been reported as *C.terrestris* in Ehime Prefecture exhibited the specific traits of *C.deflexa* (Fukuoka and Hayakawa 2016). These misidentifications can be partially attributed to the difficulty in distinguishing between the two species on the basis of their morphological traits. All the key traits observed in this study can be plastic in *C.deflexa*. Therefore, depending on the plant condition and number of observations, one may be unable to find any characteristic traits even from an actual *C.deflexa* specimen. For proper identification of *C.deflexa* and *C.terrestris*, it is necessary to observe as many flowers and fruits as possible from a single plant to determine whether it has plastic traits expected in *C.deflexa*. If it is not possible to observe a specimen with a sufficient number of flowers or fruits, it is recommended to allow the specimen to grow for a while or use DNA sequencing to determine the species.

The distribution of *C.terrestris* and *C.deflexa* in Japan needs to be investigated in the future. However, *C.terrestris* is found in only limited areas of the Kanto Region (Kanagawa and Ibaraki Prefectures), whereas *C.deflexa* is widely found in Kanto and western Japan. Both species have been found in Kanagawa and Ibaraki Prefectures, so their distribution seems to overlap in the Kanto Region. *Callitrichedeflexa* was once reported in Taiwan in the 1970s (Hsu and Yang 1976, Yang and Hsu 1998, Chen et al. 2013) and it was first collected from Okinawa, Japan, in 1999, suggesting that it may have colonised Japan from the southern region. *Callitrichedeflexa* may have colonised multiple times, as the specimens examined in this study showed genetic variation in the *matK* sequence (Table 3).

The results of this study suggest ecological differences between the two species. In Japan, *C.terrestris* plants were found only from April to June (Katsuyama and Kawasumi 1999, Suppl. material 1). In fact, in the habitat in Ibaraki Prefecture, *C.terrestris* was not present in February, but was thriving in May and it then disappeared in August (Table 1). In contrast, *C.deflexa* was often collected in winter (Table 1), indicating that it is cold-tolerant and able to overwinter in Japan. Additionally, *C.deflexa* was found in August and September in Japan (Table 1), suggesting that it is tolerant to high summer temperatures. Therefore, the differences in the life cycles of *C.deflexa* and *C.terrestris* in Japan may contribute to their different dispersal patterns.

This study revealed that the plant specimens identified as *C.terrestris* in previous developmental studies were, in fact, *C.deflexa* (Koga et al. 2021, Doll et al. 2021b, Doll et al. 2021a). In a previous study, we analysed plasticity in leaf development (heterophylly) by using *C.palustris* and chose “*C.terrestris*” as a phylogenetically close, but non-heterophyllous relative for comparison of leaf development (Koga et al. 2021). Although the material was found to be *C.deflexa* in this study, *C.deflexa* is also a suitable, albeit not optimal, terrestrial non-heterophyllous plant. We also used *C.deflexa* as “*C.terrestris*” for studies of stomatal development and evolution in *Callitriche* (Doll et al. 2021a, Doll et al. 2021b). However, in the present study, we confirmed that *C.terrestris* exhibits stomatal development characteristics similar to those of *C.deflexa*: stomata differentiate on both sides of the leaf and stomatal lineages may undergo amplifying division (Fig. 2). It is notable that these features were confirmed in a terrestrial species of *Callitriche* that belongs to a clade different from previously examined *C.deflexa* and *C.japonica*. Thus, the conclusions of previous studies were not invalidated by the misidentification.

## Supplementary Material

XML Treatment for Callitriche deflexa

XML Treatment for Callitriche terrestris

56DE0AFD-78A0-51D8-AEDE-867C629ED2A2 10.3897/BDJ.12.e115142.suppl1 Supplementary material 1 Species occurrences Data type Table Brief description A list of examined herbarium samples. File: oo_930177.xlsx https://binary.pensoft.net/file/930177 Hiroyuki Koga, Yuki Doll, Wataru Ohnishi, Horokazu Tsukaya

9DE81924-2E6A-5462-B5A6-016991834680 10.3897/BDJ.12.e115142.suppl2 Supplementary material 2 Accession numbers of sequences used in phylogenetic analysis Data type Table Brief description Sequence information of the phylogenetic analysis. File: oo_930178.xlsx https://binary.pensoft.net/file/930178 Hiroyuki Koga, Yuki Doll, Wataru Ohnishi, Horokazu Tsukaya

## Figures and Tables

**Table 1.** Plants collected for this study

Name	Species	Location	Collected month	Habitat	Specimen ID	Notes
HN2	*C.deflexa*	Nishinomiya, Hyogo	Sep 2015	Farm	00256641(TI)	Used in previous studies (Doll et al. 2021a, Doll et al. 2021b, Koga et al. 2021), 5th generation plant of inbred line was examined
OH1	*C.deflexa*	Hirakata, Osaka	Sep 2015	Farm	00256643(TI)	1st generation plant of inbred line was examined
FF2	*C.deflexa*	Fukuoka, Fukuoka	Feb 2020	Plantation	00256647(TI)	1st generation plant of inbred line was examined
IbTkb1	*C.deflexa*	Tsukuba, Ibaraki	Feb 2022	Green house	00256639(TI)	
KHd1	*C.deflexa*	Hodogaya, Kanagawa	Mar 2022	Plantation	00256640(TI)	Also found in Aug 2022
TKoi1	*C.deflexa*	Koishikawa, Tokyo	Mar 2022	Plantation	00256646(TI)	
KKmz1	*C.terrestris*	Kamimizo, Kanagawa	May 2022	Playground	00256644(TI)	Locality consistent with NA0112762(KPM), not found in Aug 2022
KHsm1	*C.terrestris*	Hashimoto, Kanagawa	May 2022	Garden	00256645(TI)	Locality consistent with NA0112764(KPM), not found in Aug 2022
IbTcu1	*C.terrestris*	Tsuchiura, Ibaraki	May 2022	Park	00256642(TI)	Not found in Mar and Aug 2022

**Table 2.** SNVs in *rbcL*.

Specimen	Position in ORF								Accession	Source
	159	165	504	555	657	793	1072	1168		
LC177674.1 *C.deflexa*	A	T	C	C	G	A	A	T	LC177674	Ito et al. (2017)
FF2	.	.	.	.	.	.	.	.	LC765361	
HN2	.	.	.	.	.	.	.	.	LC765362	
KHd1	.	.	.	.	.	.	.	.	LC765365	
OH1	.	.	.	.	.	.	.	.	LC765368	
TKoi1	.	.	.	.	.	.	.	.	LC765369	
IbTkb1	.	.	.	.	.	.	.	.	LC765364	
KHsm1	.	.	.	T	A	.	C	C	LC765366	
KKmz1	.	.	.	T	A	.	C	C	LC765367	
IbTcu1	.	.	.	T	A	.	C	C	LC765363	
AF248024.1 *C.terrestris*	.	.	.	T	A	.	C	C	AF248024	Philbrick and Les (2000)
AF248012.1 (C.deflexa*) *likely misidentification	G	C	T	T	.	G	C	C	AF248012	Philbrick and Les (2000)

**Table 3.** SNVs in *matK*.

Specimen	Position in ORF																Accession	Source
	448	562	564	586	598	599	600	625	661	699	876	966	1045	1098	1193	1250		
LC176831.1 *C.deflexa*	A	A	G	G	G	A	A	T	T	A	T	A	A	T	T	C	LC176831	Ito et al. (2017)
FF2	.	.	.	.	.	.	.	.	.	.	.	.	.	.	.	.	LC765346	
HN2	.	.	.	.	.	.	.	.	.	.	.	.	.	.	.	.	LC765347	
KHd1	.	.	.	.	.	.	.	.	.	.	.	.	.	.	.	.	LC765350	
OH1	.	.	.	.	.	.	.	.	.	.	.	.	.	.	.	.	LC765353	
TKoi1	.	.	.	.	.	.	.	.	.	.	.	.	.	.	.	.	LC765354	
IbTkb1	.	.	.	.	.	.	.	.	.	.	.	.	.	.	.	.	LC765349	
KHsm1	T	T	A	T	T	T	C	G	C	G	A	C	T	A	G	T	LC765351	
KKmz1	T	T	A	T	T	T	C	G	C	G	A	C	T	A	G	T	LC765352	
IbTcu1	T	T	A	T	T	T	C	G	C	G	A	C	T	A	G	T	LC765348	
NA0208310 (KPM)	NA	T	.	.	.	.	.	.	.	NA	NA	NA	NA	NA	NA	NA	LC765676	
NA0203540 (KPM)	NA	T	.	.	.	.	.	.	.	NA	NA	NA	NA	NA	NA	NA	LC765677	
NA0202813 (KPM)	.	.	.	.	.	.	.	.	.	.	.	.	.	.	.	.	LC765359	
NA1004372 (KPM)	NA	T	A	T	T	T	C	G	C	NA	NA	NA	NA	NA	NA	NA	LC765355	
NA1104918 (KPM)	NA	T	A	T	T	T	C	G	C	NA	NA	NA	NA	NA	NA	NA	LC765356	
NA0112762 (KPM)	NA	T	A	T	T	T	C	G	C	NA	NA	NA	NA	NA	NA	NA	LC765357	
NA0162807 (KPM)	NA	T	A	T	T	T	C	G	C	NA	NA	NA	NA	NA	NA	NA	LC765360	
NA0216556 (KPM)	NA	T	A	T	T	T	C	G	C	NA	NA	NA	NA	NA	NA	NA	LC765358	
